# Training Working Memory in Adolescents Using Serious Game Elements: Pilot Randomized Controlled Trial

**DOI:** 10.2196/games.8364

**Published:** 2018-05-23

**Authors:** Wouter J Boendermaker, Thomas E Gladwin, Margot Peeters, Pier J M Prins, Reinout W Wiers

**Affiliations:** ^1^ Addiction Development and Psychopathology (ADAPT)–Lab Department of Psychology University of Amsterdam Amsterdam Netherlands; ^2^ Department of Interdisciplinary Social Science Utrecht University Utrecht Netherlands; ^3^ Department of Psychology & Counselling University of Chichester Chichester United Kingdom

**Keywords:** cognitive function, memory, video games, motivation

## Abstract

**Background:**

Working memory capacity has been found to be impaired in adolescents with various psychological problems, such as addictive behaviors. Training of working memory capacity can lead to significant behavioral improvements, but it is usually long and tedious, taxing participants’ motivation to train.

**Objective:**

This study aimed to evaluate whether adding game elements to the training could help improve adolescents’ motivation to train while improving cognition.

**Methods:**

A total of 84 high school students were allocated to a working memory capacity training, a gamified working memory capacity training, or a placebo condition. Working memory capacity, motivation to train, and drinking habits were assessed before and after training.

**Results:**

Self-reported evaluations did not show a self-reported preference for the game, but participants in the gamified working memory capacity training condition did train significantly longer. The game successfully increased motivation to train, but this effect faded over time. Working memory capacity increased equally in all conditions but did not lead to significantly lower drinking, which may be due to low drinking levels at baseline.

**Conclusions:**

We recommend that future studies attempt to prolong this motivational effect, as it appeared to fade over time.

## Introduction

### Background

Psychological problems that occur during adolescence are often associated with deficiencies in self-regulation [1-3]. For example, working memory capacity (WMC [4]) and inhibition are often impaired in adolescents with Attention-Deficit/Hyperactivity Disorder (ADHD [2,5]). During adolescence, youngsters typically start experimenting with risky behaviors [6]. For example, consumption of alcohol usually starts in early adolescence and often at a much earlier age than is legally allowed [7]. Heavy use at this age can lead to school dropout [8] and can escalate into more severe problems later on, such as substance dependence or addiction.

Heavy drinking in youth has previously been associated with suboptimal cognitive control functions (eg, [9,10]). According to Dual Process Models of Addiction (eg, [11]), addictive behaviors emerge when an individual fails to self-regulate the impulsive reactions that develop with heavy substance use. The effects of these reactions on cognitive processing are termed cognitive biases, which can be detected using various implicit measures [11,12]. Both inhibition [13,14] and WMC [15,16] have been found to be weaker in heavy drinking youth, thus leading to an imbalanced cognitive system [17]. As such, early intervention programs aimed at training cognitive control may play an important role in keeping these mental problems at bay. WMC, or the ability to adaptively update and monitor representations in working memory [2], has been considered the most central of cognitive control functions [18]. WMC has been the target of many training studies aiming to improve WMC, with some moderate successes in children with relatively weak WMC [19], such as children with ADHD (for review, see [20]; but see [21]). Increasing WMC has also led to reduced drinking in problem drinkers with strong automatic positive associations with alcohol [22], as well as to positive changes in symptoms of anxiety, increased inhibitory control, and reduced attention to threat in adolescents [23].

Despite its efficacy in specific adolescent groups, motivation is an important moderator of cognitive training efficacy [11,24]. As cognitive training paradigms can be very long and tedious, with as many as 25 separate sessions for WMC training (eg, [25,26]), motivation to train is likely to decline during training, which may impact the training’s efficacy. Incorporating game elements into the cognitive training paradigms may help adolescents to persevere, as such elements may be better at grasping and retaining adolescents’ attention and increasing their motivation to complete the training [27].

There have been several attempts to gamify cognitive training paradigms. For example, Prins et al [28] developed an elaborate game world called Braingame Brian around multiple evidence-based executive function training principles. Positive training effects with this gamified training have been found in obese children [29] and children with ADHD [26]. However, Braingame Brian is primarily aimed at primary school–aged children and may be perceived as too childish by adolescents. For this reason, we developed the City Builder game [30], which is specifically aimed at training cognitive functions and retraining substance-related cognitive biases in adolescents.

### Objectives

The City Builder game was designed as a so-called game-shell [31], where the user receives points for doing well on the training task. The training task itself was only minimally adjusted from the original evidence-based training paradigm to fit the game environment. The points collected during training could be spent during periods of play time in between the training blocks [30]. Besides the game elements, an element of alcohol-related context was also added to the training task by briefly showing a picture of alcohol during the encoding phase of the task. As this picture could be more distracting to heavier drinkers, it could potentially make the training a little more challenging for this group.

This pilot study describes the results of 10 sessions of alcohol-related WMC training using the City Builder game. We compared 3 conditions (all including the alcohol-related context): the gamified WMC training using the City Builder game (henceforth referred to as the *gamified* condition); a nongamified WMC training (the *standard* condition); and a nongamified placebo training, not expected to improve WMC (the *placebo* condition). The primary focus of the pilot study was on how the game could help to motivate adolescents to continue training over the course of 10 sessions. Adolescents in the gamified condition were expected to show a higher motivation to train, compared with adolescents in both nongamified conditions, as measured by explicit ratings and the time spent on training. In addition, the training was expected to increase WMC, relative to placebo. As a secondary outcome, we looked at potential transfer effects of WMC training to drinking behavior, where participants were expected to drink less alcohol after the training. Furthermore, as an exploratory analysis, the potential influence of the alcohol picture on performance was analyzed, and it was expected that heavier drinkers in the sample would make more errors following the alcohol picture.

Finally, a practical problem that can occur in an experimental comparison of a training task with and without game elements is that although all participants complete the same assessments after the training, only those in the gamified condition have been rewarded during training. As these participants may have been getting used to being rewarded for their effort, the lack of rewards in the posttraining assessment could negatively affect their motivation, and in effect their performance, potentially distorting the assessment of the training effect in an unwanted way [31]. Because it is difficult to prevent this influence in an experimental research design, motivation for doing the pre- and posttraining assessments was also evaluated using self-report questions.

## Methods

### Participants

Participants were 84 adolescents from a high school in the Netherlands aged between 13 and 16 years (mean age 13.7 [SD 0.7] years; 40% [34/84] boys). Participants trained during normal school hours in 14 groups of 6 students. They were randomly assigned to 1 of the 3 training conditions stratified for age, gender, and school class. Participants in each group were allocated to the same condition (as a form of clustered randomization) to prevent them from comparing the gamified and nongamified versions among each other. There were 24 students (4 groups) in the placebo condition, 30 students (5 groups) in the standard WMC training condition, and another 30 students (5 groups) in the gamified WMC training condition. The training took place in 2 cohorts: 7 groups (2 placeboes, 3 standard WMC training, and 2 gamified WMC training) finished training before Christmas break; the other 7 groups started after Christmas. The second cohort filled in an additional questionnaire assessing motivation to train after each session. Due to personal reasons, 3 students (2 from the placebo and 1 from the standard WMC training condition) dropped out during the study. The study’s target sample size was between 25 and 30 participants per condition, which was based on similar studies [26,27] using a gamified working memory task. The study was approved by the Ethics Committee of the University of Amsterdam (Protocol number 2012-COP-2449).

### Design and Procedure

Before the study, parental consent was obtained for each adolescent, and at baseline, adolescents were informed about the training procedure and the reward for participation, which was a maximum of 15 euros, consisting of 5 euros for doing the baseline and posttraining assessments and an additional 1 euro for each completed training session. The training itself was not presented to the participants as an alcohol intervention; rather, it was presented as a new “computer training” that was to be tested, which could help them to gain more “mental control” over their behavior, such as (excessive) alcohol use. To keep the students motivated to continue training in all conditions, it was announced that the training money was only awarded when a minimum of 8 training sessions were completed. The training was done on university laptops in groups of 6 adolescents, whereas the assessment sessions, which were the same in all 3 conditions, were done in groups of 12 students on school personal computers. After the baseline assessment, participants performed 10 daily training sessions on school days during the next 2 weeks. When a training session was missed because of an important school activity, an extra training session was planned for a total of 10 training opportunities per participant. Finally, there was a posttraining assessment session.

### Training

#### Standard Working Memory Capacity Training

This training was based on the Chessboard task by Dovis et al [27], but with the inclusion of several alcohol pictures. The alcohol picture was intended to slightly distract participants, with an expected greater effect on participants who drink more alcohol, as their attention can be biased toward alcohol pictures [32,33], which can affect task performance on a working memory task [34]. Participants were presented with a 4×4 grid of green and blue squares (each 120×120 pixels large, presented in a chessboard pattern) that lit up in a specific sequence of 3 or more squares. The instruction was to remember this sequence, then mentally reorder the squares to reproduce first all green squares, and then all blue squares, in the order in which they appeared. To ensure reordering was necessary in each trial, each sequence showed at least one blue square before one or more green squares. During trials, the sequence length was first announced in the center of the screen for 1500 ms. Each square then lit up for 1500 ms, with a 1000 ms interval between squares, until the current number of squares was shown. A 540×540 pixel image of a beverage containing alcohol was shown for 600 ms during one of the intersquare intervals (selected randomly). Different sets of 10 unique pictures were used for this purpose during each training session. All alcohol stimuli were taken from the Amsterdam Beverage Picture Set [35]. To prevent the use of memory strategies, the mouse cursor was invisible during the trials. After each trial, there was always feedback about whether the answer was correct, followed by a self-paced button to go to the next trial. During feedback, a progress bar also indicated how far they were during the session. When 2 consecutive trials were answered correctly, the next sequence length was increased by one square. Similarly, when 2 consecutive trials were answered incorrectly, the next sequence length became one square shorter, with a minimum of 3 squares. Each training session lasted approximately 30 min and consisted of a minimum of 40 trials, with a first 3-min break after the first block of 20 trials and a second 3-min break after the second block of 20 trials. After the second break, participants received the option to continue with another block of training trials or wait for 5 min before going back to class collectively with the other participants in the group.

#### Placebo Working Memory Capacity Training

This version was exactly the same as the standard WMC training, except that the sequence length was always kept at 3 to prevent a training effect while presenting a visually similar experience (cf [22]). As the overall duration of the task was shorter because of keeping the sequence length at a low level, participants in the placebo condition did a minimum of 50 trials per session (25 per training block).

#### Gamified Working Memory Capacity Training

**Figure 1:**
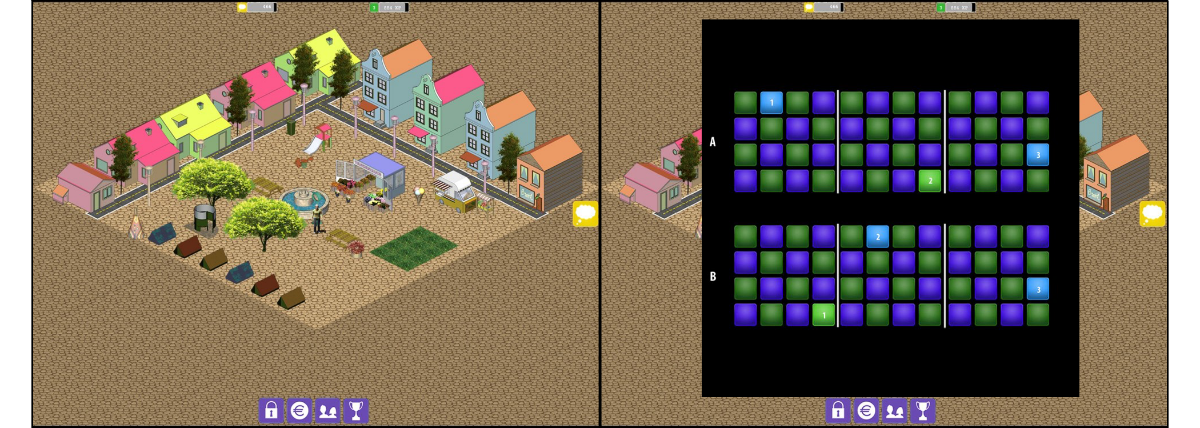
The City Builder game. Left pane: the game screen; Right pane: the working memory capacity (WMC) training task is presented overlaying the game screen. During instructions, the game is shown in the background (as pictured); when the trials start, the background blacks out entirely.

This version was also similar to the standard WMC training but was embedded within a game context, the City Builder game ([30]; see Figure 1). As in the other conditions, each training session started with a block of training trials, but in the gamified WMC training condition, participants received points for correct trials. These points were saved up until the break and could then be spent as game money to buy houses, roads, trees, and other objects to build a virtual city. A social element was included in the game by letting participants view the cities built by other players, which they were also allowed to rate with a “thumbs up.” After the break (which lasted exactly 3 min), the game automatically reverted to another training block, followed by the second break. As the final training block did not include any play time, the extra collected points could only be spent during the next training session.

As shown in Table 1, the breaks between the training blocks were introduced to match the time between participants in all conditions. All conditions were given 3 optional activities during the breaks, which they could switch between however they liked: either continue training, read a book or magazine, or spend the time in silence (cf [36]), but no phones or Internet use were allowed. Only participants in the gamified WMC training condition were allowed to use this time to play the game. These alternative ways to spend the break were intended to be potentially interesting alternatives for playing the game, so that those who did chose to continue with the game indeed did so because they liked doing so, rather than being bored. Training trials done during the break did not count toward the minimum of trials during the fixed training blocks. A final block of optional bonus trials was included as an additional behavioral measure of motivation to train. The same options were provided as during the breaks, but now also those in the gamified WMC training condition were not allowed to play the game.

**Table 1:** Procedure during training sessions.

Version of working memory capacity training	Standard	Placebo	Gamified
Training block 1 (9 min)	20 trials	25 trials	20 trials
Break 1 (3 min)	Continue training, read magazine, or enjoy break in silence	Continue training, read magazine, or enjoy break in silence	Continue training, read magazine, enjoy break in silence*,* or *play the game*^a^
Training block 2 (9 min)	20 trials	25 trials	20 trials
Break 2 (3 min)	Continue training, read magazine, or enjoy break in silence	Continue training, read magazine, or enjoy break in silence	Continue training, read magazine, enjoy break in silence*,* or *play the game*
Optional extra training block (5 min)^b^	Continue training, read magazine, or enjoy break in silence	Continue training, read magazine, or enjoy break in silence	Continue training, read magazine, or enjoy break in silence

^a^During the first session, participants in the gamified working memory capacity training condition always started the first break with a 1-min introduction to the game.

^b^During the last session, the second break lasted for 8 min, and the extra training block was omitted, as there was no next session to spend the bonus points in.

### Measures

#### Working Memory Capacity Assessment

WMC was assessed using the Self-Ordered Pointing Task (SOPT; [37]). In the SOPT, the participant is shown a set of pictures with the instruction to click on a picture they have not clicked on before. Then the pictures in the set are shuffled, and the instruction is repeated, until the number of responses equals the number of pictures presented in the set. The current version used increasingly larger sets of pictures and alternated between sets of pictures of concrete objects (eg, ball, umbrella) and sets of pictures of abstract objects (eg, lines and figures), in the following order: 4 concrete (practice), 6 concrete, 6 abstract, 8 concrete, 8 abstract, 10 concrete, 10 abstract, 12 concrete, and finally 12 abstract pictures. This was done to gradually increase the difficulty of the task to avoid a ceiling effect. The primary outcome measure of the SOPT was the total number of correct responses over all test blocks, that is, a score between 8 and 72, with a higher score indicating better WMC (for reliability and validity, see [38]).

#### Motivation to Train

Besides the number of bonus trials done per session (ie, during both breaks as well as in the final, optional training block) as a behavioral measure of motivation, 2 self-report questions were also added in the second cohort: “How much were you looking forward to this task?” and “How much did you like this task?,” both scored on a 10-point scale ranging from 1 (not at all) to 10 (very much). After the training, participants were asked about their previous game experience, as well as how much fun they thought the training had been, on a 5-point Likert scale from 1 (a lot of fun) to 5 (very boring); how difficult they thought the training had been, on a 5-point Likert scale from 1 (very difficult) to 5 (very easy); and how often they would continue doing the training if it would be made available at home, on a 5-point Likert scale from 1 (never) to 5 (very often).

#### Alcohol Use

As heavy drinking does occur at this age in the Netherlands [7], a brief personal interview version of the Alcohol Timeline Followback (TLFB) Procedure [39,40]) was used to measure alcohol consumption per day over the past 10 days. The personal interview was used to offer participants a more private and secure environment, compared with the computer room. In addition, potential alcohol-related problems were assessed with the Alcohol Use Disorder Identification Test (AUDIT [41]), the Rutgers Alcohol Problem Index (RAPI18 [42]), and the Five-Shot Questionnaire [43]. The AUDIT includes 10 multiple-choice questions about alcohol consumption and alcohol-related problems. The overall score ranges between 0 and 40, with a score of 8 or higher indicating an increased risk of alcohol-related problems. The RAPI18 is an 18-item questionnaire for assessing problem drinking, specifically among adolescents. Each item concerns a statement about the frequency of an alcohol-related problem occurring during the past year, with scores on a 4-point Likert scale ranging from 0 (never) to 3 (more than 5 times). The Five-Shot Questionnaire contains 5 multiple-choice items about alcohol use. The maximum score is 7, with a score over 2.5 indicating alcohol misuse or alcohol dependence.

#### Additional Baseline Measures

To check for baseline differences in intelligence quotient (IQ), a subselection of 30 items from Raven Standard Progressive Matrices (RPM [44]) was assessed. Baseline differences in reward sensitivity were checked using the Dickman Impulsivity Inventory [45], which contains 23 true or false questions divided over 2 subscales, and the Behavior Inhibition System/Behavior Approach System scale [46,47], which consists of 20 Likert scale items over 4 subscales. Finally, basic family structure, family drinking habits, and parental social economic status were also assessed.

### Statistical Analyses

Before running the analyses, all dependent variables were screened for univariate outliers (scores removed more than 3 SDs from the group mean), which resulted in the exclusion of 2 outliers on the AUDIT, 1 on the Five-Shots Questionnaire, 4 on the TLFB, 1 on the SOPT sum score, 2 on the RAPI18, 1 on the SOPT, 1 on the BAS Fun seeking, and 2 on the BAS Reward responsiveness subscales. Due to technical problems, the data of 4 participants at baseline, TLFB data for 3 participants, and RPM data for 1 participant were lost. All analyses were thus performed on the remaining number of participants.

The hypothesized effects of training condition over time were ascertained through the use of factorial repeated measures analyses of variance, using condition as a between-subjects factor (with 3 levels: standard, placebo, and gamified), and time as a within-subjects factor (with 2 levels: before and after the training). Motivation was compared on several measures using regular analyses of variance (or nonparametric variants thereof, in those cases where one or more statistical testing assumptions were violated), as well as a growth model analysis on the number of bonus trials done during each session. Finally, an exploratory analysis of variance was performed using the percentages on specific squares following the alcohol picture.

## Results

### Baseline and Missing Data

Due to various reasons (eg, illness), some participants missed one or more sessions but were allowed to continue training. Five participants, however, did not complete the full assessments and were therefore excluded from the relevant prepost analyses. In total, 29 participants completed the full training in the gamified WMC training condition; 27 in the standard WMC training condition and 20 in the placebo condition. Levels of drinking were very low at baseline. The average sum score on the AUDIT was 1.2 (SD 2.3), with 52 participants having a sum score of 0, and 0.4 (SD 1.1) on the RAPI18. Therefore, it was decided to include these 2 long-term measures again after training to make sure this finding was stable. This was the case. There were no baseline differences in age, gender, IQ, impulsivity, or WMC between conditions (all *P* values >.05).

### Effects of Training

There was a main effect of time on WMC as measured with the SOPT sum score, *F*_1,72_=6.033, *P*=.02, *η*_p_^2^=0.077, but no effect of training condition, *F*_2,72_=0.052, *P*=.95, *η*_p_^2^=0.001 (see Table 2). When an inclusion threshold of participants who had completed at least 8 out of 10 training sessions (cf 20 of 25 sessions in [22]) was used as a cut off for the effects analyses, resulting in the exclusion of 2 participants in the gamified WMC training condition, 3 in the standard WMC training condition, and 2 in the placebo condition, these effects did not change. There was no training effect on alcohol consumption as measured with the TLFB over time, *F*_1,62_=1.410, *P*=.24, *η*_p_^2^=0.022.

### Motivation

Table 3 features several measures of motivations by group. There was a slight trend that suggests more participants preferred to have the game at home compared with the nongamified versions. The standard WMC training was rated as less fun to do, Kruskal-Wallis *H*_2_)=10.093, *P*=.006, compared with both the gamified (Mann-Whitney’s *U*=233.0, *z*=3.145, *P*=.002, *r*=−.413) and the placebo version (*U*=410.5, *z*=2.128, *P*=.03, *r*=.301). Motivation to do the SOPT assessment increased over time in the nongamified conditions, but it decreased in the gamified WMC training condition, a difference that was significant, *F*_2,28_=7.363, *P*=.003, *η*_p_^2^=0.345. Post hoc comparisons using the Tukey HSD test indicated that the mean score for the gamified WMC training condition (−0.6 [SD 1.2]) was significantly lower than both the standard WMC training (1.3 [SD 1.8]) and the placebo condition (1.0 [SD 0.8]). A similar pattern of results was observed for the change in the level of fun on the SOPT, but these did not reach significance. Finally, there was a difference in the average number of training sessions completed between conditions, where adolescents in the gamified WMC training condition completed significantly albeit slightly more sessions on average than participants in the 2 nongamified conditions.

**Figure 2:**
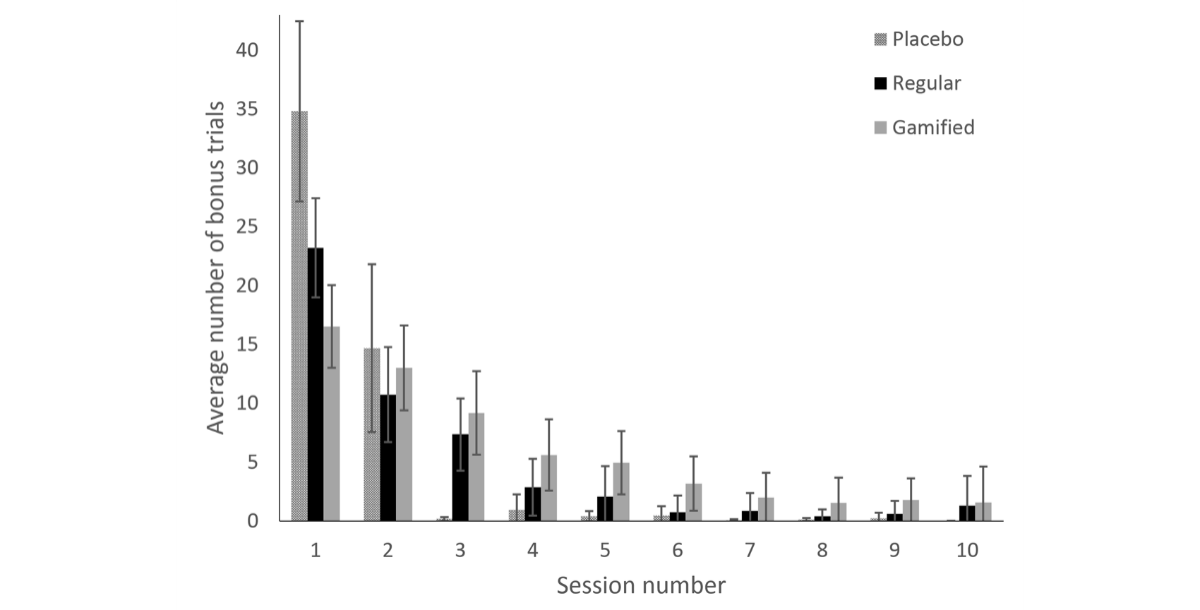
Average number of bonus trials per session. Error bars indicate 95% CI.

As another measure of motivation to train, we looked at the total number of bonus trials done during each session (ie, during both breaks as well as in the final, optional training block), where we numbered the sessions per participant (see Figure 2 and Table 4). For this analysis, we used a multiple-step approach. As the count variable (number of bonus trials) had a skewed distribution, but not all sessions had many zeros, a Poisson distribution was used rather than zero inflation (cf [14]).

Robust Maximum Likelihood was used as an estimator to account for the nonnormality. The first step taken was a confirmatory factor analysis (CFA) on the total number of bonus trials during each session (cf [48]). As session 1 showed much higher numbers of bonus trials in all conditions, compared with the following sessions, the CFA did not converge when session 1 was included, and it was therefore excluded from the analysis. The resulting CFA on sessions 2 through 10 showed that all factor loadings were significant. Due to the nature of the Poisson model, using numerical integration, no standardized factor loadings are available. The second step involved looking at the overall effect of condition on the latent session factor, which was significant: *B*=.444, SE=0.088, *P*<.001, indicating more bonus trials were done in the gamified WMC training condition compared with the other conditions.

In the final step, we looked at change over time using a growth model of sessions 2 through 10, again with the bonus trials count variables as latent growth indicators. Several models were compared, first constraining groups to be equal or not (ie, assuming there were or there were no group differences), and subsequently constraining only the slopes to be equal or not (ie, assuming there were or there were no differences in the decrease of bonus trial counts), and the intercepts to be equal or not (ie, assuming there were or there were no baseline differences in bonus trial counts). The best model fit in terms of Akaike Information Criterion (AIC [49]), as well as the Bayesian Information Criterion (BIC [50]), was found for the model with free (decreasing) slopes, but with constrained (equal) intercepts for the standard and gamified WMC training conditions, AIC=2758; BIC=2782. In this model, the placebo training’s intercept is at 0.667, whereas both the standard and gamified WMC training’s intercepts are at 1.219; slope coefficients are −2.855 for the placebo, −1.782 for the standard, and −0.859 for gamified WMC training. Note that due to the nature of the count model used here, these coefficients do not represent the actual number of bonus trials but should rather be interpreted relative to each other, for example, the decrease is much steeper in the placebo condition compared with the gamified WMC training condition.

**Table 2:** Training outcomes by group.

Measure	Standard	Placebo	Gamified	Total
SOPT^a^ sum score pretraining, mean (SD)	55.4 (4.5)	55.1 (4.8)	56.2 (4.5)	55.6 (4.6)
SOPT sum score posttraining, mean (SD)	57.4 (5.3)	57.3 (4.3)	55.9 (4.8)	56.8 (4.9)
TLFB^b^ sum score pretraining, mean (SD)	0.3 (0.6)	0.2 (0.5)	0.1 (0.2)	0.2 (0.5)
TLFB sum score posttraining, mean (SD)	0.3 (0.7)	0.1 (0.2)	0.0 (0.2)	0.1 (0.4)

^a^SOPT: Self-Ordered Pointing Task.

^b^TLFB: Timeline Followback; shows the number of standardized drinks during the week before the assessment.

**Table 3:** Motivations by group.

Measure		Standard	Placebo	Gamified	Total	*P* value
**Questions assessing motivation**						
	How much fun was the training? (mean [SD])^a^	3.7 (0.7)	3.2 (0.9)	3.1 (0.7)	3.3 (0.8)	.006^b,c^
	Would you like to have the training at home? (yes; absolute [%])	2 (7)	0 (0)	5 (17)	7 (9)	.10
	How often would you train at home? (mean [SD])^d^	1.4 (0.7)	1.3 (0.5)	1.7 (0.8)	1.5 (0.7)	.13^b^
	How much were you looking forward to this task (the SOPT)? (mean [SD]^e,f^)	1.3 (1.8)	1.0 (0.8)	−0.6 (1.2)	0.4 (1.6)	.003^c^
	How much did you like this task (the SOPT)? (mean [SD]^e,f^)	1.1 (1.8)	0.5 (1.1)	−0.1 (1.8)	0.4 (1.7)	.21
Number of training sessions completed (mean [SD])		8.8 (1.1)	8.4 (1.1)	9.1 (0.8)	8.8 (1.0)	.04^g^

^a^5-point Likert scale from 1 (a lot of fun) to 5 (very boring).

^b^Nonparametric Kruskal-Wallis test was applied due to violation of normality.

^c^*P*<.01.

^d^5-point Likert scale from 1 (never) to 5 (very often).

^e^Mean (SD) of change score. Change score is defined as the difference between the pre- and posttraining assessment scores.

^f^10-point grade from 1 (low) to 10 (high).

^g^*P*<.05.

**Table 4:** Average number of bonus trials per session.

Session	1	2	3	4	5	6	7	8	9	10
Placebo, mean (SD)	34.8 (19.2)	14.7 (17.8)	0.2 (0.4)	1.0 (3.2)	0.4 (1.1)	0.5 (1.9)	0.1 (0.2)	0.1 (0.3)	0.2 (0.8)	0.0 (0.0)
Standard, mean (SD)	23.2 (11.8)	10.7 (11.1)	7.3 (8.4)	2.9 (6.6)	2.0 (7.0)	0.8 (3.8)	0.9 (3.9)	0.4 (1.4)	0.6 (2.5)	1.3 (3.4)
Gamified, mean (SD)	16.5 (9.8)	13.0 (10.0)	9.2 (9.9)	5.6 (8.5)	4.9 (7.5)	3.2 (6.5)	2.0 (5.8)	1.5 (5.7)	1.8 (4.7)	1.6 (4.7)

**Table 5:** Error percentages on specific squares.

Measure		Standard	Placebo	Gamified	Total	*P* value
**Including placebo condition (N=84), mean (SD)**						
	Error percentage on squares directly following the alcohol picture	24.2 (5.8)	5.8 (3.8)	24.7 (4.8)	19.1 (9.8)	<.001^a,^^b^
	Error percentage on squares not directly following the alcohol picture	24.3 (5.9)	6.8 (4.3)	24.2 (5.2)	19.3 (9.5)	<.001^a,b^
	Ratio of errors directly following the alcohol picture over those that do not	1.00 (0.08)	0.85 (0.13)	1.03 (0.09)	0.97 (0.12)	<.001^a,b^
**Without placebo condition (N=60), mean (SD)**						
	Error percentage on squares directly following the alcohol picture	24.2 (5.8)		24.7 (4.8)	24.5 (5.2)	.33^a^
	Error percentage on squares not directly following the alcohol picture	24.3 (5.9)		24.2 (5.2)	24.2 (5.5)	.66^a^
	Ratio of errors directly following the alcohol picture over those that do not	100.3 (8.2)		103.0 (8.7)	101.6 (8.5)	.22
	Average sequence length^c^	5.5 (0.8)		5.6 (0.7)	5.5 (0.7)	.41

^a^Nonparametric Kruskal-Wallis test, which was applied due to violation of normality.

^b^*P*<.001.

^c^The average number of squares shown per trial.

To determine the influence of the alcohol picture during the encoding phase of the training trials, we looked at the percentage of errors made specifically on squares that directly followed the alcohol picture versus the error percentage on squares that did not directly follow the alcohol picture. Overall, error percentages were different between the training conditions, but this was mainly because in the placebo condition, all sequences had exactly 3 squares, and thus fewer errors were made. When this condition was excluded, the standard and gamified WMC training conditions did not differ (see Table 5). The average sequence length also did not differ between the standard and gamified WMC training conditions (the placebo condition was not included as all sequences had exactly 3 squares). As the level of drinking was very low in this sample, no relationships between error percentage and alcohol consumption were found (all *P* values >.05).

## Discussion

### Principal Findings

In this pilot study, we investigated the beneficial effects of a serious game environment on adolescents’ motivation to do cognitive training. Although no relevant differences were found in the primary outcome measure (WMC), several interesting findings were obtained regarding motivation to train. First, the self-reported motivation questions posed after the training was completed showed mixed results, with participants only having a slight preference against the standard WMC training. This may indicate that participants did not like the game more than they liked the placebo WMC training, but it can also mean that they merely lost interest over time. Other than the nongamified training versions, the gamified WMC training, being presented as a game, likely increased participants’ expectations of its entertainment value. If the game then did not fully satisfy these expectations over the 10 sessions of training, this may have influenced the motivation assessment after the training. As such, it is advisable to assess motivation to train at multiple points in time to see if there might be an initial effect that fades over time. This can be achieved with a behavioral measure of motivation, such as the number of training trials done beyond the minimum amount required. This number was found to be higher in the gamified WMC training condition than in the nongamified conditions, but it also declined over time in all conditions.

Regarding the bonus trial analysis, the fact that the first session showed a much higher numbers of bonus trials in all conditions, compared with the following sessions, may actually make sense from a theoretical standpoint. Given that during the first session, all versions of the training were new to the participants, when the option to do extra trials was first presented, it may have been curiosity rather than motivation that drove participants to do some bonus trials. From the second session forward, though, this option was no longer novel, suggesting that actual motivation to train would have taken over.

It should be noted that the wish to spend the points collected through training by playing the game during the breaks may have limited the time available for doing bonus trials. This may have inadvertently led to an underestimation of the motivation to train. Relatedly, the number of bonus trials may have been skewed a little due to the fact that, on average, bonus trials in the placebo condition were shorter than those in the active training conditions. This might explain the initial peak in the placebo condition in session 1, while also underscoring the fact that the decline in sessions 2 and 3, which is attributed to motivation, is also most notable in this condition. As we unfortunately did not record the time spent on doing bonus trials or playing the game, the number of bonus trials was the only behavioral measure of motivation we were able to analyze. Future studies should therefore consider also looking at the time spent on bonus trials and playing the game as additional behavioral measures of motivation.

A theoretical explanation for the declining motivational effect found, in terms of fewer bonus trials done per session over time, could be that the points awarded during training may be acting primarily as extrinsic motivators [51]. Although the points have value in that they can almost immediately be turned into game assets, they are arguably less fun to collect on their own. This means that although playing the game in between the training blocks may tap into some intrinsic motivation for the participant, the training itself remains limited to extrinsic motivators. As extrinsic motivators are known to suffer from diminishing returns [52], it is likely that they provide less motivation to train over time, which can explain the decline in bonus trials [31]. Future research could focus on determining the specific intrinsic or extrinsic motivational value of each game element by comparing them separately. Although the design used in this pilot study is not suited for such a comparison, as each condition either included all or none of the game elements, identifying those game elements that specifically tap into participants’ intrinsic motivation may help to make motivation to train last longer.

### Strengths and Limitations

In line with previous motivational results [36,53-55], the gamified WMC training version was found to motivate adolescents to train more intensively over the course of the 10 training sessions, compared with the nongamified versions. Finding ways to motivate adolescents to sustain a high training performance is very important as long as these training paradigms remain long and tedious. Interestingly, the beneficial effects found by Dovis et al [55] were the combined result of a gamified WMC training and additional systematic external reinforcement by training coaches. Although this pilot study did not use coaches, it could be argued that a combination of motivational game elements and external reinforcement might give better motivational results.

The second motivational finding concerns participants’ motivation to perform well on the study’s main cognitive outcome measure: the pre- and posttraining WMC assessments (SOPT). Although WMC was found to increase over time in all training conditions, which could indicate a practice effect, where participants’ performance increased due to having done the task before, motivation to complete the task had increased after the training in the nongamified conditions but had decreased in the gamified WMC training condition. This finding is in line with our hypothesis that the rewarding nature of the gamified WMC training condition may negatively affect motivation to complete assessment tasks afterward. Although it is unclear if, and to what degree, this motivational effect may have influenced the assessment of the actual training gain, it does have important implications for future research aiming to validate serious games, compared with their nonrewarding, original counterparts. Incorporating the assessment task in the game and having a mini-assessment at the start of every training session (cf [56]) is one option to prevent decline in motivation for the postassessment in the gamified WMC training condition. However, this may also intensify the entire training program by prolonging its overall duration.

The results presented in this paper do have to be interpreted with some caution because of several limitations. First, no training effects were found on drinking behavior; however, alcohol use was very low at baseline in this sample. As it obviously could not get much lower through training, no inferences on the effects of (gamified) cognitive training on drinking behavior should be made based on this study. It would be interesting for future intervention research to include adolescents with cognitive deficits and at risk for problematic alcohol use [57]. Second, when comparing the active training conditions, there were no discernable effects of the alcohol pictures presented during training trials on the percentage of errors made during these trials, nor did they affect the average sequence length. When the active training conditions were compared with the placebo condition, the latter showed a notably lower percentage of errors on squares directly following the alcohol picture. This could be due to the easiness of trials in the placebo condition, so that presentation of a distractor resulted in a more optimal level of arousal, but further research is necessary to disentangle this effect. Although the alcohol pictures may have inadvertently introduced a priming effect, which was not assessed separately, they were presented in the same manner in all conditions, and no effects on drinking were found. Nevertheless, future studies that incorporate alcohol pictures in their WMC training should consider assessing, for example, attentional bias toward alcohol before and after exposure, especially if a future training study is done in heavier drinkers. Third, despite the fact that we did find an increase in WMC over time, this did not go beyond the level found in the placebo group. Several studies report optimal cognitive and behavioral training results (eg, reduced alcohol intake [22]) with around 15 to 25 sessions of training [20,58], rather than the 10 sessions presented here (but see the study by de Voogd et al [59], who found significant training results using an emotional WMC training over 8 sessions). The fact that the game’s benefit to participants’ motivation to train had already faded over 10 sessions underscores the need for a solution for the motivational aspect of the training. Future studies are thus encouraged to design motivating game elements aimed at adolescents that keep the training fun for at least that many sessions. Finally, although each group of 6 participants was randomized into the same condition, some school classes still included groups of students allocated to different conditions. Therefore, it cannot be ruled out that conversations between students about the differences between the conditions may have had an influence on motivation. To prevent this, future studies should include a larger sample which allows for cluster randomization using full classes or, preferably, full schools.

### Conclusions

Despite these limitations, to the best of our knowledge, this study is the first to demonstrate that WMC training in adolescents can benefit from the use of game elements by increasing motivation to train. It follows that the challenge for future research will be in trying to prolong this effect, for example, by making bigger, more immersive games that last longer (although this is quite a challenge, even in commercial gaming). By closely monitoring the levels of motivation throughout the study, as well as by managing participants’ expectations about the entertainment value of the training, which may still be an important factor in determining the training outcome, more insight may be acquired into the specific effectiveness of the use of game elements in cognitive training. Finally, future research could also apply gamified WMC training in specific at-risk groups, such as adolescents who have specific difficulties with traditional training approaches due to attention- or motivation-related problems. Moderation analyses can then be used to reveal individual differences in the effectiveness of the gamified training, identifying those who could benefit the most from these motivational features.

## Abbreviations

ADHD: attention-deficit/hyperactivity disorder
AIC: Akaike information criterion
AUDIT: Alcohol Use Disorder Identification Test
BIC: Bayesian information criterion
CFA: confirmatory factor analysis
IQ: intelligence quotient
RAPI18: Rutgers alcohol problem index
RPM: Raven standard progressive matrices
SOPT: self-ordered pointing task
TLFB: Alcohol Timeline Followback
WMC: working memory capacity
